# Cognitive testing of the Children’s Palliative Outcome Scale (C-POS) with children, young people and their parents/carers

**DOI:** 10.1177/02692163241248735

**Published:** 2024-05-06

**Authors:** Lucy Coombes, Debbie Braybrook, Daney Harðardóttir, Hannah May Scott, Katherine Bristowe, Clare Ellis-Smith, Lorna K Fraser, Julia Downing, Myra Bluebond-Langner, Fliss EM Murtagh, Richard Harding

**Affiliations:** 1 King’s College London, Florence Nightingale Faculty of Nursing Midwifery and Palliative Care, Cicely Saunders Institute of Palliative Care, Policy and Rehabilitation, London, UK; 2 Royal Marsden NHS Foundation Trust, London, UK; 3 International Children’s Palliative Care Network, Kampala, Uganda; 4 University College London, Louis Dundas Centre for Children’s Palliative Care, London, UK; 5 Wolfson Palliative Care Research Centre, Hull York Medical School, University of Hull, Hull, UK

**Keywords:** Patient-reported outcome measurement, cognitive interviewing, palliative care, children, outcome assessment

## Abstract

**Background:**

The Children’s Palliative Outcome Scale (C-POS) is being developed using best methodological guidance on outcome measure development, This recommends cognitive testing, an established method of item improvement, prior to psychometric testing.

**Aim:**

To cognitively test C-POS within the target population to establish comprehensibility, comprehensiveness, relevance and acceptability.

**Design:**

Cross-sectional cognitive interview study following COnsensus-based Standards for the selection of health Measurement INstruments (COSMIN) methodology and Rothrock guidance on outcome measure development. Cognitive interviews were conducted using ‘think aloud’ and verbal probing techniques.

**Setting/participants:**

Children 5–⩽17 years old with life-limiting conditions and parents/carers of children with life-limiting conditions were recruited from 14 UK sites.

**Results:**

Forty-eight individuals participated (36 parents; 12 children) in cognitively testing the five versions of C-POS over two to seven rounds. Content and length were acceptable, and all questions were considered important. Refinements were made to parent/carer versions to be inclusive of non-verbal children such as changing ‘share’ to ‘express’ feelings; and ‘being able to ask questions’ to ‘having the appropriate information’. Changes to improve comprehensibility of items such as ‘living life to the fullest’ were also made. Parents reported that completing an outcome measure can be distressing but this is anticipated and that being asked is important.

**Conclusion:**

Cognitive interviewing has facilitated refinement of the C-POS, especially for non-verbal children who represent a large proportion of those with a life-limiting condition. This study has enhanced the face and content validity of the measure and provided preliminary evidence for acceptability for use in routine practice.

**What is already known about this topic?**

- The Children’s Palliative Outcome Scale is being developed using methodological guidance on patient-reported outcome measure development.
- Cognitive testing of patient-centred outcome measures is recommended to ensure relevance, comprehensiveness and comprehensibility.

**What this paper adds**

- This paper demonstrates that it is possible to develop a single patient-centred outcome measure for use with children of all ages with a wide range life-limiting conditions and their families.
- Minor adaptations were required to ensure comprehension and relevance to children of different chronological and developmental ages. A proxy version is required for non-verbal children with adaptations to some items required to ensure relevance.
- Children with life-limiting conditions are able and willing to participate in cognitive interview studies, even if they have communication difficulties or developmental delay.

**Implications for practice, theory or policy**

- This paper highlights the importance of cognitive testing of patient-centred outcome measures, a step of measure development that is not always conducted.
- It is essential that parents of children with life-limiting conditions are given the opportunity to complete outcome measures, even if some items cause distress.

## Background

It is estimated that each year 21 million children and young people worldwide (‘children’) with life-limiting or life-threatening (‘life-limiting’) conditions require input from palliative care services.^1^ Life-limiting conditions are those for which there is no hope of cure, and from which children will die. Life-threatening conditions are those for which curative treatment is feasible but may fail.^2^

Previous work has been conducted in sub-Saharan Africa to develop a patient-centred outcome measure for use in paediatric palliative care.^3–5^ Development of the African Children’s Palliative Outcome Scale (C-POS) began before recent accepted guidance on patient-reported outcome measure development was proposed and the content is informed by the African healthcare context.^6^ To address this gap, a UK version of the C-POS has been developed following COSMIN (COnsensus-based Standards for the selection of health Measurement INstruments) and Rothrock psychometric methodological guidance on patient-centred outcome measure (PCOM) development.^6–8^ Item generation and content validity were informed by a qualitative semi-structured interview study,^9,10^ a systematic review to inform optimal recall period, response format and administration mode,^11^ a Delphi survey to achieve consensus on priority outcomes and an item generation meeting.^12^ This has resulted in five versions of C-POS – two parent/proxy versions (child <2 years and ⩾2 years) and three child versions (5–7, 8–12 and 13–17 years or cognitive equivalent). The concepts explored are the same in each version, with simpler language for younger/less cognitively able children (Table 1). Child versions are named after planets to avoid stigma associated with using chronological age in children with life-limiting conditions, many of whom have developmental delay. Following item generation, it is recommended that item improvement processes are conducted to ensure relevance, comprehensiveness and comprehensibility and to reduce response error.^6,13^

**Table 1.** C-POS items.

Child symptom and concern items (self-reported or proxy-reported)	Parent/carer items
Pain	Getting enough sleep
Other symptoms	Access to information about child’s condition
Being able to ask questions	Support needed to care for child
Being able to undertake usual activities	Support to plan for future care
Worry	Impact of child’s condition on family
Sharing feelings	Support to plan future care
Being able to do things you enjoy	
Living life to the fullest	

Cognitive interviewing is an established method of item improvement of PCOMs, recommended by COSMIN.^7^ Conducting cognitive interviews has been shown to yield three times more problematic items than identified by using item non-response.^14^ Participants are asked to complete the measure while ‘thinking aloud’ with or without verbal probing.^15^ ‘Think aloud’ cognitive interviews require participants to verbalise their thought processes while answering questions, and the interviewer intervenes as little as possible.^13^ This can provide insight into the type of information participants retrieve from memory.^16^ Verbal probing is often used in conjunction with ‘think aloud’ techniques, to locate problems with questions^15,17^ and assess comprehension of a measure.^16^

This study aimed to use cognitive interviews to test C-POS for comprehensibility, comprehensiveness, relevance and acceptability within the target population.

## Methods

Cross-sectional cognitive interview study following COSMIN and Rothrock guidance.^6,7^ This study is reported according to the Cognitive Interview Reporting Framework.^18^

### Setting

Participants were recruited from ten NHS sites and four children’s hospices within England, Scotland and Northern Ireland.

### Sampling and recruitment

Inclusion criteria: Children 5–⩾17 years living with a life-limiting condition and parents/carers (‘parents’) of children 0–⩾17 years with a life-limiting condition who can speak and/or read English

Exclusion criteria: Children unable to communicate wishes themselves or via their parent, enrolled in another study, clinically unable or unwilling to provide consent/assent. Parents unable or unwilling to provide consent.

We aimed to test each version with at least seven participants as per COSMIN recommendations,^7^ with any amendments being made after four interviews. If amendments were required, we aimed to test the revised version with three further participants, with the final version being tested with at least three participants. Participants were purposively sampled to ensure maximum variation in children’s age and diagnosis.

### Data collection

Interviews were conducted face-to-face or virtually via Microsoft Teams, dependent on participant preference and COVID-19 guidance. Prior to interview, participants completed a consent process and short demographic questionnaire. Participants were then given a demonstration of the ‘think aloud’ technique and encouraged to take part in a practice task, whereby they were asked to count the number of windows in their house while thinking aloud.^15^ This task helped to build a rapport between the interviewer and participant. Interviews were conducted using the ‘think aloud’ technique with concurrent verbal probing.^15,19,20^ A Supplemental topic guide was used to ensure pre-scripted probes regarding item wording, recall and response format. After all C-POS items had been cognitively tested participants were asked questions regarding their acceptability, relevance and comprehensiveness. This included asking whether any items were inappropriate and should be left out, whether any important items were missing and should be added to C-POS, and whether the number of items and time taken to complete the measure was acceptable. Spontaneous probes were used to explore any hesitations or difficulties.^14^

**Figure 1.**
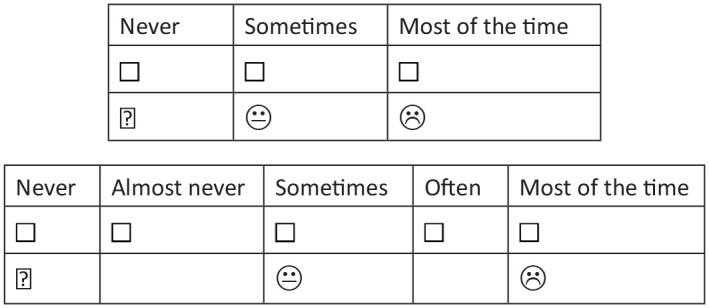
Response formats tested.

Version selection for child participants was guided by developmental age, allowing the child and parent to choose the most appropriate version. Previous work highlighted challenges in ascertaining at what chronological/developmental age children can reliably use longer recall periods and more complex response formats.^11^ In all children >8-years-old, both a 3- and 5-point response scale format was tested, along with recall periods of the past week and yesterday and today (Figure 1). Children <8-years-old tested a 3-point response format and recall of yesterday and today. The use of emojis to anchor responses was informed by evidence from previous work^12^ with a smiling emoji representing the positive outcome for the item. All child versions of C-POS had eight questions.

Proxy versions of C-POS had a recall period of the past week and a 5-point Likert response format (Never, Almost never, Sometimes, Often, All of the time). Proxy versions contained eight questions about the child (identical concepts to self-report items), and five about the family.

Interviews were conducted by LC, DB and HS (experienced qualitative researchers) and DH (new to qualitative research). Interviews were audio recorded, transcribed verbatim and pseudonymised.

### Data analysis

Analysis followed the five steps of Framework analysis recommended by National Centre for Social Research for analysing cognitive interview data: familiarisation, constructing a thematic framework, indexing and sorting, charting and mapping/interpretation.^20–23^ Data from all interviews was summarised into a single data set using Microsoft Excel. A matrix was created for each C-POS item; each row of the matrix representing an individual participant, and each column an area of investigation.^20–23^ Areas of investigation were based on Tourangeau’s four-stage model of survey response – comprehension, retrieval, judgement and response^21^ and Willis’ coding system for classifying questionnaire problems.^22^ Interviews were analysed independently by two members of the team. Data were reviewed after each item had been tested with four participants and findings were discussed in regular research team meetings. Any problems identified and potential changes to C-POS were discussed and agreed as a group.

### Ethics and consent

Ethical approval was granted by the Bloomsbury ethics committee (HRA: 21/LO/0282). Participants over 16 years provided written informed consent. Those with parental responsibility provided written informed consent for participants under 16 years. Those under 16 years were given the opportunity to provide written assent.

## Results

### Sample characteristics

Forty-eight individuals (36 parents; 12 children) participated between June 2021 and April 2022 (Table 2). Diagnoses are classified according to ICD-10-chapter headings to preserve participant anonymity, as many life-threatening conditions are rare.^24^

**Table 2.** Participant demographics.

Parent/carer of child testing <2 years C-POS version (*n* = 10)	
Gender	7 female: 3 male
Age (years) (mean; range)	34.9 (30–41)
Age of child (months) (mean; range)	26.1 (2.5–108)^a^
Diagnosis of child	1 Cancer
	5 Congenital
	1 Metabolic
	3 Neurological
Ethnic background	2 Asian/Asian British
	8 White British
Length of interview (min) (mean; range)	74.2 (37–144)
Parent/carer of child testing >2 years C-POS version (*n* = 26)	
Gender	22 female: 4 male
Age (years) (mean; range)	45.4 (32–60)
Age of child (years) (mean; range)	10.7 (2–17)
Diagnosis of child	4 Cancer
	6 Congenital
	3 Metabolic
	13 Neurological
Ethnic background	1 Black/Black British
	1 Other
	24 White British
Length of interview (min) (mean; range)	60.6 (25–121)
Children testing 5–7 years C-POS version (*n* = 3)	
Gender	1 female: 2 male
Age (years) (mean; range)	11 (7–16)^a^
Diagnosis	1 Congenital
	2 Neurological
Ethnic background	3 White British
Length of interview (min) (mean; range)	32.7 (26–42)
Child testing 8–12 years C-POS version (*n* = 6)	
Gender	6 female
Age (years) (mean; range)	11.5 (10–13)^a^
Diagnosis	2 Cancer
	1 Congenital
	3 Neurological
Ethnic background	1 Other
	5 White British
Length of interview (min) (mean; range)	38.0 (13.5^b^–83)
Young person testing 13–17 years C-POS version (*n* = 3)	
Gender	1 female; 2 male
Age (years) (mean; range)	15.0 (14–16)
Diagnosis	1 Cancer
	1 Congenital
	1 Neurological
Ethnic background	3 White British
Length of interview (min) (mean; range)	53.5 (39.0–69.5)

a Version was tested according to developmental not chronological age.

b One participant only completed three questions and was too unwell to continue.

## Main findings

C-POS was tested over two to seven rounds dependent on version. Interview findings and subsequent changes made to C-POS are displayed in Tables 3 and 4. All participants were able to participate in the cognitive interview process after the practice task. Some children under 8 years needed direction and explanation from a parent during the first few questions. Understanding of the ‘think aloud’ task then improved. Participants 5- to 7-years-old (or cognitive equivalent) frequently needed an explanation of items prior to responding.

**Table 3.** Main findings from cognitive interviews with children and young people.

Item number	C-POS item	Comprehension	Retrieval	Judgement	Response	Changes made to response format	Changes made to question
1	Hurt (5–12 years) Pain (13–17 years)	Good comprehension in those over 8-years-old^a^. Younger children^a^ understood after a verbal explanation. Question answered in terms of frequency, rather than severity or impact pain had on day-to-day life.	The majority of those 8- to 17-year^a^ could recall the past week. Some interpreted this to mean since Monday or the start of the weekend. Some 5- to 7-year^a^ struggled with yesterday and could only report on the current day.	Those 5- to 7-year^a^ needed some help from a parent to integrate their thoughts into a response. Those 8- to 17-year^a^ had no difficulties.	Some concerns that those 5- to 7-year^a^ chose the response they thought the interviewer wanted to hear. Emojis made choosing easier. 8–12 years^a^ showed variability in ability to use a 5-point response format. Those 13- to 17-year^a^ all preferred and could use 5-point response format.	‘Most of the time’ replaced with ‘All of the time.	Quantifiers removed from beginning of question (How much, how often etc) to allow severity and impact to be reported in addition to frequency.
2	Other problems with your body	Well-understood by those 8- to 12-years old^a^. Young children sometimes needed a verbal explanation. One participant included emotional problems. Question answered in terms of frequency, rather than severity or impact symptoms had on day-to-day life.	The majority of those 8- to 12-year^a^ could recall the past week. One 5- to 7-year-old^a^ could only remember the current day.	No problems integrating thoughts into a response.	Those 5- to 7-year^a^ could use the 3-point response format. About 8–12 years^a^ showed variability in ability to use a 5-point response format. Those 13- to 17-year^a^ all preferred and could use 5-point response format.	As above	Quantifiers as above
3	Worry	Good comprehension in all participants except one 5- to 7-year-old^a^.	All those 8- to 12-year^a^ could recall the past week. One participant 5- to 7-year^a^ could not understand the recall period yesterday and today and discussed salient events in the recent past.	All but one participant (5–7 years^a^) could integrate their thoughts into an appropriate response.	As above	As above	Quantifiers as above
4	Sharing feelings	Good comprehension. One 5- to 7-year-old^a^ required a verbal explanation from parent to understand the question.	All participants could retrieve the information required. All those 8- to 12-year^a^ except one participant could recall the past week.	No problems integrating thoughts into an appropriate response.	As above	As above	Quantifiers as above ‘Sharing feelings’ changed to ‘been able to talk to people’ in 5- to 7-year-old^a^ version
5	Being able to do the things you usually would	Good comprehension.	One child 5- to 7-year^a^ could only recall the current day. One child 8- to 12-year^a^ thought back to the start of the weekend (interview was a Wednesday).	Several children 5- to 7-year-old^a^ wanted clarity regarding whether usual things were those done currently, pre-diagnosis or pre COVID-19 pandemic.	As above	As above	Quantifiers as above
6	Being able to do things that are fun (5–7 years) Being able to do things you enjoy (8–17 years)	Good comprehension – all could explain the difference between ‘usual activities’ and ‘fun things/things you enjoy’.	Those 5- to 7-year^a^ could recall yesterday and today. Those 8- to 17-year^a^ could recall the past week, although one referred back to start of the week (interview was mid-week).	No major problems integrating thoughts into a response. One participant asked same question as above regarding pre/post diagnosis and COVID-19 pandemic.	As above	As above	Quantifiers as above
7	Enjoying life as much as possible (5–7 years) Living life to the fullest (8–17 years)	Younger children understood ‘enjoying life as much as possible’. For children aged 8–12^a^ only half understood ‘living life to the fullest’. The rest preferred the 5- to 7-year^a^ question. Children 13- to 17-year^a^ could comprehend and explain what living life to the fullest meant to them.	One child 5- to 7-year^a^ answered generally without relating response to required recall period. Those 5- to 7-year^a^ could recall the past week. One 8- to 12-year-old^a^ expressed a preference for a recall of yesterday and today.	No problems integrating thoughts into a response.	As above	As above	Quantifiers as above ‘Living life to the fullest’ changed to ‘Enjoy life as much as possible’ in 8- to 12-year-old^a^ version
8	Being able to ask important questions	No problems with comprehension in those 5- to 7-year^a^. Younger children needed a verbal explanation of what the question meant.	One 5- to 7-year-old^a^ could only recall the current day. Those 12- to 17-year^a^ could recall the past week.	No problems integrating thoughts into a response.	As above	As above	Quantifiers as above

a Or cognitive equivalent.

**Table 4.** Main findings from cognitive interviews with parent/proxies.

Item number	C-POS item	Comprehension	Retrieval	Judgement	Response	Changes made to response format	Changes made to question
*Items about the child*							
1	Pain	No problems	No problems	No problems. Those with children <2 years or non-verbal children could all formulate a response based on child’s behavioural cues.	No problems. Answered in terms of frequency, rather than severity and distress.	Extra response option added ‘Not appropriate to my child’	Quantifiers removed from beginning of question (how much and how many) to allow severity and impact to be reported in addition to frequency.
2	Other symptoms	No problems	No major problems. A few participants spoke of answering based on events of approximately the past week, as if your child is unwell days all merge together.	No problems arriving at a response.	No problems	As above	Quantifiers as above
3	Crying more than usual (<2 years) Affected by worry (>2 years)	No problems	No problems	<2 years – Participants had difficulty judging which episodes of crying to include when formulating a response that is, crying due to frustration, tantrums, pain, falling over, hunger. Also, participants struggled to judge what ‘more than usual’ meant as crying often becomes less frequent as babies get older. >2 years – parents of children who were non-verbal had difficulty formulating a response. This question worked much better when wording was changed to ‘expressed anxiety or worry’.	All response options understood. Participants struggled to choose an option due to issues with judgement.	As above	Quantifiers as above. <2-years changed to ‘displayed signs of worry or anxiety over the past week for example, by being more irritable, sad, clingy or withdraw’. >2 years changed to ‘expressed anxiety and worry’
4	Express feelings (<2 years) Opportunity to share feelings (>2 years)	No problems.	No problems	Some difficulty in judging which response to pick for children who were non-verbal or who had developmental delay as they could not verbally share feelings. The question worked better when it was changed to ‘opportunity to express feelings’.	All response options understood. This question worked better for parents of children who could not express feelings in a meaningful way when the ‘not appropriate to my child option’ was added.	As above	Quantifiers as above. >2 years changed to ‘opportunity to express feelings’
5	Being able to do the things child usually would	One participant struggled to articulate the difference between this item and the following item about things the child enjoys doing. All other participants could describe and understand the difference.	No problems	No problems formulating a response	No problems	As above	Quantifiers as above.
6	Being able to do things child enjoys	No problems	No problems	Most participants no problems formulating a response. One participant questioned whether the item was appropriate for a 5-month-old baby and struggled to formulate a response.	No problems	As above	Quantifiers as above.
7	Reach full potential (<2 years) Live life to the fullest (>2 years)	The majority of participants whose child was <2 years understood ‘full potential’ to mean in comparison to a healthy child. Only two interpreted it as being in the context of their child’s condition. >2 years – Participants interpreted ‘live life to the fullest’ as comparing their child to healthy children.	No problems	<2-year version – Some difficulty due to problems comprehending the question. Some participants were unsure what their child’s ‘full potential’ was and whether this was related to physical development or cognitive ability. >2 years – Amending to ‘live life to *their* fullest’ worked better and allowed participants to talk about the child in the context of their condition.	<2 years – Response options were understood but most participants struggled to choose one due to comprehension and judgement issues. This improved when ‘not appropriate to my child’ as added as a response option. >2 years no problems.	As above	Quantifiers as above. Both versions changed to ‘Has your child been able to live life to *their* fullest’
8	Communicate needs (<2 years) Ask questions (>2 years)	No problems	No problems	<2 years No problems >2 years – participants struggled to formulate an answer if their child was non-verbal. This improved when the wording was changed to ‘had the appropriate information for them about their condition’.	No problems	As above	Quantifiers as above. >2 years changed to ‘had the appropriate information for them about their condition’
*Items about the family*							
9	Information about child’s illness	No problem. Several participants suggested the term ‘illness’ should be changed to ‘condition’.	No problems recalling the past week. Some participants felt that the recall period should be longer for this question, as the need for information reduces after initial diagnosis	Most participants had no issues with judgement. Two struggled to decide what type of information they should include when formulating their response for example, medication management, condition-specific.	Most participants understood the responses. One suggested a dichotomous yes/no response option would be better.	No changes	Quantifiers as above. ‘Illness’ changed to ‘condition’.
10	Support needed to provide care	No problems	No problems	No problems. Most participants discussed medical, psycho-emotional and practical support.	No problems	No changes	Quantifiers as above.
11	Planning for care	Participants interpreted this item to be about planning child’s immediate care, such as respite stays and home care. When the term ‘future’ was added this changed to understanding it to be about anticipatory planning for the future.	No problems	No problems with judging a response. When the term ‘future’ was added to the question, participants spoke about advance care planning and transition.	No problems	No changes	Quantifiers as above. Changed to ‘planning for future care’ as question was intended to ask about advance care planning.
12	Impact of child’s illness on family	No problem. Several participants suggested the term ‘illness’ should be changed to ‘condition’.	No problems	No problems	No problems	No changes	Quantifiers as above. Term ‘illness’ replaced with ‘condition’
13	Parent/carer tiredness/fatigue	No problems	No problems	Most participants felt fatigue and tiredness were two different concepts and should be asked about separately. Judgement was improved when question was amended to ‘able to get enough sleep’.	Difficult to choose a response due to judgement issues regarding using the terms fatigue and tiredness in the same question. Response was improved when question was changed to asking about sleep.	No changes	Quantifiers as above. Changed to ‘able to get enough sleep’.
14	Access to psychological and emotional support (question added to final round of cognitive testing)	No problems	No problems	Participants discussed both formal psychological and emotional support, as well as informal support from family and friends.	No problems	No changes	No changes

### Findings related to all versions

In round 1 participants responded to items in terms of frequency, so quantifiers (how much, how often) were removed from the stem of questions, so they began with ‘Have you been affected by’. . .. In subsequent rounds, participants responded in terms of how much they had been affected by a symptom or concern.

The original response format in the child versions was amended so that ‘most of the time’ became ‘always’. Children felt this fitted with ‘never’ at the other end of the scale and wanted definitive ‘always’ and ‘never’ response options.

### Child and young person versions

The child versions of C-POS were tested over two rounds.

#### Comprehension

In round 1, two items posed comprehension problems. Children 5- to 12-years-old did not understand the term ‘live life to the fullest’. This was amended to ‘enjoy life as much as possible’. Those 5- to 7-years-old found difficulty understanding the term ‘sharing feelings’. This was changed to ‘been able to talk to people’. These were tested in round 2 and were understood well.

‘I: *And so, when we ask about sort of live your life to the fullest, what are you thinking about in those things?*

P: I don’t know.’ (CHI8-12d)

#### Retrieval

All participants 13- to 17-year-old could use a recall period of the past week. They preferred this option to ‘yesterday and today’.

*‘I think again it [yesterday and today] isn’t a long enough timescale so I do prefer the past week’ (CHI13-18a)*

Retrieval ability varied in 8- to 12-year-olds, with some only being able to recall yesterday and today, and some responding to things that had happened since the start of the week or weekend, rather than the past 7 days. Those 5- to 7-years-old sometimes struggled with the concept of yesterday, reporting only about the current day. No changes were made to the recall period as the intention is to use the version most appropriate to the child’s developmental ability.

#### Judgement

Participants 5- to 7-years old initially needed some support from their parent to formulate a response. The ability to respond independently improved as they moved through the measure. Items regarding ‘usual activities’ and ‘things you enjoy’ posed difficulties for some participants. There was uncertainty about whether the benchmark for responding should be in those 8- to 17-years old: activities undertaken pre-diagnosis, current activities or activities they were able to undertake before the COVID-19 pandemic.

*‘Child: Have you been able to do the things that you usually would, over the past week? Like, have you been able to do the things that you usually would, before having your disease? Or like, as in, like you, that you usually do, with your disease, but you’ve just had another thing happened?*

*Interviewer: That’s for you to tell me. What does it make you think of? So with that phrase, things you usually would, what do you think of with that question?*

*Child: Um, [pause] I think before my disease. Because I don’t know, like, when it comes to mind like, I would normally like, like last year I would be, you know, going out, and stuff, because it’s like, almost spring. Uh, but now I can’t really do that, because of COVID and everything. And obviously, I can’t risk getting infections or anything.’ (CHI8-12e)*

#### Response

All participants 13-years and over could use a 5-point response format and expressed preference for this option over the 3-point format.

*‘Um, because there’s more options. Easier to find one that. . . makes sense.’ (CHI13-18c)*

There was variability in those 8- to 12-year, with some being able to use a 5-point response format and some managing better with a 3-point format.

Participants could describe in which circumstances they would choose specific response options, suggesting understanding of how to use these:

*‘Because, in my week, I get, I have Monday to Friday of radiotherapy, so that’s every single day, and then I get Saturday and Sunday off. So then I think, I get like, a good few days of doing things that I really enjoy. But then, if I think about yesterday or today, that could be a Sunday or a Saturday, and I would say, Yeah, that’s often. Then I would say, if it was a Tuesday or Wednesday, I would say sometimes.’ (CHI8-12e)*

#### Acceptability, relevance and comprehensiveness

All children found the content and number of questions acceptable. All items were reported to be important. No additional items were identified. The emojis were well-liked and made selecting a response option easier.

*‘All of them [the questions] were important to me.’ (CHI5-7c)*

*‘Thank you. It’s been good. It’s been good to have someone ask questions that are, like, asking about how I actually feel about everything. Not just, are you in pain? Do you need some paracetamol?’ (CHI8-12e)*

### Parent/carer versions

The CPOS version for parents of children ⩾2 years was tested over seven rounds: the version for parents of children <2 years over four rounds.

#### Comprehension

Two items posed comprehension problems. When responding to the items regarding ‘reaching full potential’ (<2 years) and ‘being able to “live life to the fullest”’ (>2 years), participants compared their child to healthy children. It was intended that this question was answered within the context of their child’s life-limiting condition. Amending this to ‘live life to *their* fullest’ across both proxy versions allowed greater comprehension. The item ‘having support planning for care’ was intended to ask whether participants felt supported in advance care planning decisions. However, this was interpreted to mean planning for day-to-day care needs. Amending this to ‘planning for future care’ allowed the item to be understood as intended.

#### Retrieval

Participants had no problems with retrieval. Some suggested a longer recall for the ‘information needs’ item as these needs are often higher at diagnosis. This was not changed as recall period is the same for all items.

#### Judgement

There were several issues with judgement, particularly for parents of non-verbal children. ‘Sharing feelings’ and ‘asking questions’ were amended to ‘express feelings’ and ‘having appropriate information’ to be more inclusive of the range of life-limiting conditions. Judgement difficulties were also identified with the ‘crying more than usual’ item. Participants were unsure whether to include crying due to frustration, temper or falling over in their response. They also had difficulty deciding what ‘more than usual’ meant. This item was amended to ‘displayed signs of worry or anxiety’ which improved judgement.

*‘Well. . . cried, it’s probably almost never, but he is getting more upset and frustrated with things. But that’s, not crying, that’s different. So probably almost don’t. Um no, I think, if we’re looking at just proper crying. . .’ (PAR0-2d)*

The item ‘tiredness and fatigue’ also posed difficulty. These were felt to be two different concepts requiring different responses. The item was changed to ‘been able to get enough sleep’.

*‘So, I think tiredness and fatigue, sometimes can mean a bit different. Like I think tiredness can, like, you could be like lack of sleep. And fatigue could just be like exhaustion. . ..’(PAR0-2a)*

#### Response

The 5-point Likert scale was easy to use. Some participants suggested that a ‘not appropriate to my child’ option was added for those who felt that their child had no understanding of, or could not articulate, concepts such as worry and information needs.

#### Acceptability, relevance and comprehensiveness

Measure length was acceptable. Participants found some questions upsetting to answer (particularly ‘planning future care’), however none felt any items should be removed.

*‘You can’t not ask those questions because they’re important questions.’ (PAR0-2g)*

*‘Some of them [questions] could be upsetting but that goes back to what I said, this is just an upsetting situation.’ (PAR2-18l)*

Several participants suggested adding an additional item regarding psychological or emotional support for the family. They felt this was not incorporated in the item regarding support needed to care for their child. This was added as item 14.

Finally, some participants expressed that when completing a PCOM about their child, they expected it to be emotive at times. They felt that all items should be included. They also felt that the process of cognitive interviewing made the questions more emotive than they would be in a clinical scenario.

*‘I think that anyone who’s got a child in palliative care, who’s agreed to be in a study about palliative care, should know that they might get a little bit upset while answering questions cos it’s sad, you know?’ (PAR2-18l)*

*‘But it’s because of the way that we’re having to discuss our thought processing about why we’re – that is – that – that makes it more emotive in this scenario than it might have otherwise done’ (PAR2-18q)*

### Final C-POS versions

All versions of C-POS were tested in their final format as per COSMIN recommendations.^7^ Final C-POS versions are shown in Table 5. Recall period and response format for each version are informed by the results of cognitive testing. Younger children require a short recall and simple response format and children 13- to 17-year can recall the past week and use a 5-point response format. Children 8- to 12-year showed variability in which recall and response format they could use, recalling the past week seemed to be easier than using a 5-point response scale.

**Table 5.** Final C-POS versions.

Version	Respondent	Number of questions	Recall period	Response format	Total number of participants version cognitively tested with	Number of rounds of cognitive testing	Number of participants final version tested in
A	Parent/carer of younger and non-communicative children	14 (8 about the child; 6 about the family)	Past week	5-point Likert scale + not appropriate to my child	10	4	3
B	Parent/carer of older children and those who can communicate	14 (8 about the child; 6 about the family)	Past week	5-point Likert scale + not appropriate to my child	26	7	5
Mercury	Child self-report – appropriate for children with a development age of 5–7 years	8	Yesterday and today	3-point Likert anchored with emojis	3	2	2
Saturn	Child self-report – appropriate for children with a developmental age of 8–12 years	8	Past week	3-point Likert anchored with emojis	6	2	1
Neptune	Child self-report – appropriate for children with a development age of 13–17 years	8	Past week	5-point Likert anchored with emojis	3	2	2

## Discussion

Cognitive testing of C-POS has demonstrated that for most participants items, response options and recall period were acceptable. All items in C-POS were found to be relevant for the population it is intended to be used with. Wording was amended for some items to improve comprehension and ensure they were interpreted as intended. Several questions posed difficulty for parents of non-verbal children. For example, concepts such as whether a child had been ‘able to share their feelings’ or ‘ask questions’. Amending wording to use terms such as ‘express feelings’ and ‘had appropriate information for them’ allowed C-POS to be more inclusive of the range of children with life-limiting conditions, many of whom have communication difficulties.^25^ An option for ‘not appropriate to my child’ was added for those for whom a proxy response could not be determined. All of these changes have enhanced the acceptability and comprehension of C-POS items and the measure as a whole.

C-POS length and content was acceptable to all participants. Initially many item stems began with ‘How much. . .’ or ‘How often..’. Cognitive testing identified that participants were responding to these items in terms of frequency of a symptom or concern. Removing stems so questions began with ‘Have you..’ allowed participants to answer in terms of how a symptom or concern has affected the child, which is the intention of C-POS.

Children were able to participate in cognitive testing and provide valuable insights into the design and content of C-POS. There is strong evidence that, from the age of 8-year old, children can meaningfully self-report on their own health-outcomes.^26^ There is conflicting evidence on when younger children can do this.^27^ Evidence suggests that those 4–6 years can report on concrete domains such as pain but may have difficulty with more complex or emotional aspects.^26,28^

We found that children aged 5–7 years (or cognitive equivalent) often needed an adult to explain questions to them before they could choose an appropriate response and undertake the ‘think aloud’ technique required for the study. This finding supports recommendations that when younger children are completing PCOMs they are administered with initial adult support so that more difficult concepts can be explained.^29^ Previous studies that have shown that children with life-limiting conditions prefer to complete a measure in the presence of an adult in case they have questions or need support.^12^

We found some instances of social desirability bias in children 5–7 years (or cognitive equivalent) who gave the answers they thought the researcher wanted to hear (e.g. saying they had not experienced any pain when in fact they had). Concerns have also been raised in previous studies that the use of faces in response options for children can lead to children choosing the face they like best (usually a smiling face), rather than the one that expresses how they feel.^30–32^ In our study, with further explanation, these participants were able to give appropriate responses. We found no evidence from the ‘think aloud’ responses that participants were choosing the response they liked the look of. This supports evidence that children of this age can be suggestible and may give the response they think interviewers want to hear.^28^ It is important children are encouraged to express their own thoughts when self-reporting their health outcomes, and clearly told that there are no right or wrong answers.

Children 5–7 years (or equivalent) sometimes responded to items in terms of the ‘here and now’, rather than yesterday or today. This supports evidence that young children may not always understand the difference between the past, present and future and may engage in ‘scripting’ whereby they respond about what usually happens and regularly occurring events.^26^ We found that all children were able to use a 3-point Likert response format. This contrasts with previous research which suggests that children <8 years cannot use Likert response formats and a dichotomous Yes/No response is preferable.^33^ We were keen to test whether children 5–7 years could use a 3-point Likert scale, as a dichotomous format limits the responsiveness of a measure.^33^ Future inter-rater reliability testing of C-POS will help to establish whether any additional recall and response format issues are occurring.

A small number of parents in our study found some items upsetting to answer but acknowledged that they were in a challenging and emotive situation, so this was to be expected. They did not want these items removed as they were important to address with the health care team, thus demonstrating the relevance of all items to their child’s situation. Parents also reflected that the cognitive interviewing process meant they had to think about items in more detail than they would in a clinical scenario, which could have exacerbated any distress. Parents were keen to share that they expected to experience some mild distress when taking part in a palliative care research study, and this was acceptable. This finding has implications for ethical review of future studies and supports previous findings that while caregivers can find taking part in paediatric palliative care research difficult at times, they still want to participate.^34,35^

Children and young people in our study had no suggestions for further C-POS items, suggesting that the measure is comprehensive and contains items on the most important symptoms and concerns in this population. Parents felt that an extra item was required asking about psychological and emotional support for themselves and the family. Ensuring that parents and family members of children with life-limiting conditions have adequate psychological and emotional support is essential to the well-being of the unwell child.^36^ It was initially intended that the item on ‘support needed to provide care’ would incorporate this. However, this was not how participants interpreted this item, which they comprehended as the more practical aspects of care provision, such as equipment needs and respite care. Therefore, an extra item was added to improve the comprehensiveness of the C-POS measure.

### Strengths and limitations

During this study C-POS was tested in the target population using accepted cognitive interview techniques. We have demonstrated that with support, children with life-limiting conditions can participate in cognitive testing of PCOMs. We have also added to the limited evidence on the age/developmental stage at which children can use more complex response formats and longer recall periods, highlighting that between the age of 8–12 years (or cognitive equivalent) ability to use these varies.^11^ Another strength of the study is that the C-POS version for parents of children over 2 years was tested over multiple rounds to ensure items worked for parents of non-verbal children.

Although our sample did not contain any children under 7 years old, two participants were reported by their parents to have a cognitive age of five. This may have impacted our findings, particularly regarding the comprehensibility of subjective items such as worry and the finding that children in this age group could use a 3-point Likert response format. Exploration of future psychometric data is required to affirm these findings.

The majority of participants in our study were white British, whereas the prevalence of life-limiting conditions is higher in minority ethnic groups in the UK.^24^ C-POS has initially been constructed in the English language, so those that could not speak or read English were excluded from participating, which may in part explain participant demographics.

### Next steps

Further research is required to psychometrically test C-POS followed by development of an implementation plan. Once validated, cultural adaption can be undertaken to widen the reach of C-POS, and to enable outcomes to be measured in all children with life-limiting conditions and their families.

## Conclusions

This study demonstrates the value of cognitive testing as a stage of PCOM development, as we found several issues that needed addressing. This has strengthened how problematic items are comprehended and reported. C-POS has been demonstrated to be acceptable and relevant to the target population. Comprehensiveness of the proxy-reported C-POS was enhanced by the addition of an extra item. Finally, this study demonstrates that it is feasible to measure complex multidimensional outcomes for a highly vulnerable, complex population who are often excluded from research.

## Supplemental Material

sj-docx-1-pmj-10.1177_02692163241248735 – Supplemental material for Cognitive testing of the Children’s Palliative Outcome Scale (C-POS) with children, young people and their parents/carers Supplemental material, sj-docx-1-pmj-10.1177_02692163241248735 for Cognitive testing of the Children’s Palliative Outcome Scale (C-POS) with children, young people and their parents/carers by Lucy Coombes, Debbie Braybrook, Daney Harðardóttir, Hannah May Scott, Katherine Bristowe, Clare Ellis-Smith, Lorna K Fraser, Julia Downing, Myra Bluebond-Langner, Fliss EM Murtagh and Richard Harding in Palliative Medicine

## References

[1] Connor SR Downing J Marston J. Estimating the global need for palliative care for children: a cross-sectional analysis. J Pain Symptom Manage 2017; 53: 171–177. 27765706 10.1016/j.jpainsymman.2016.08.020

[2] Together for Short Lives. Introduction to children’s palliative care, https://www.togetherforshortlives.org.uk/changing-lives/supporting-care-professionals/introduction-childrens-palliative-care/ (2019, accessed 31 May 2022).

[3] Namisango E Bristowe K Murtagh FEM , et al Towards person-centred quality care for children with life-limiting and life-threatening illness: self-reported symptoms, concerns and priority outcomes from a multi-country qualitative study. Palliat Med 2020; 34: 319–335. 32081084 10.1177/0269216319900137

[bibr4-02692163241248735] Namisango E Bristowe K Murtagh FEM , et al Face and content validity, acceptability, feasibility, and implementability of a novel outcome measure for children with life-limiting or life-threatening illness in three sub-Saharan African countries. Palliat Med 2022; 36: 1140–1153. 35656638 10.1177/02692163221099583

[5] Downing J Atieno M Powell RA , et al Development of a palliative care outcome measure for children in sub-Saharan Africa: findings from early phase instrument development. European J Palliat Care 2012; 19: 4.

[6] Rothrock NE Kaiser KA Cella D. Developing a valid patient-reported outcome measure. Clin Pharmacol Ther 2011; 90: 737–742. 21975345 10.1038/clpt.2011.195 PMC3753201

[bibr7-02692163241248735] Terwee CB Prinsen CAC Chiarotto A , et al COSMIN methodology for evaluating the content validity of patient-reported outcome measures: a Delphi study. Qual Life Res 2018; 27: 1159–1170. 29550964 10.1007/s11136-018-1829-0 PMC5891557

[8] Prinsen CAC Mokkink LB Bouter LM , et al COSMIN guideline for systematic reviews of patient-reported outcome measures. Qual Life Res 2018; 27: 1147–1157. 29435801 10.1007/s11136-018-1798-3 PMC5891568

[9] Scott HM Coombes L Braybrook D , et al Spiritual, religious, and existential concerns of children and young people with life-limiting and life-threatening conditions: a qualitative interview study. Palliat Med 2023; 37: 856–865. 36978266 10.1177/02692163231165101 PMC10227090

[10] Coombes L Braybrook D Roach A , et al Achieving child-centred care for children and young people with life-limiting and life-threatening conditions—a qualitative interview study. Eur J Pediatr 2022; 181: 3739–3752. 35953678 10.1007/s00431-022-04566-w PMC9371630

[11] Coombes L Bristowe K Ellis-Smith C , et al Enhancing validity, reliability and participation in self-reported health outcome measurement for children and young people: a systematic review of recall period, response scale format, and administration modality. Qual Life Res 2021; 30: 1803–1832. 33738710 10.1007/s11136-021-02814-4 PMC8233251

[12] Coombes L Harðardóttir D Braybrook D , et al Achieving consensus on priority items for paediatric palliative care outcome measurement: results from a modified Delphi survey, engagement with a children’s research involvement group and expert item generation. Palliat Med 2023; 37(10): 1509–1519. 37853579 10.1177/02692163231205126 PMC10657511

[13] Beatty PC Willis GB. Research synthesis: the practice of cognitive interviewing. Public Opin Q 2007; 71: 287–311.

[14] Buers C Triemstra M Bloemendal E , et al The value of cognitive interviewing for optimizing a patient experience survey. Int J Soc Res Methodol 2014; 17: 325–340.

[15] Willis GB. Cognitive interviewing: a tool for improving questionnaire design. Thousand Oaks, CA; London: Sage Publications, 2005, p.xii, 335.

[16] Knafl K Deatrick J Gallo A , et al The analysis and interpretation of cognitive interviews for instrument development. Res Nurs Health 2007; 30: 224–234. 17380524 10.1002/nur.20195

[17] Schildmann EK Groeneveld EI Denzel J , et al Discovering the hidden benefits of cognitive interviewing in two languages: the first phase of a validation study of the Integrated Palliative care Outcome Scale. Palliat Med 2016; 30: 599–610. 26415736 10.1177/0269216315608348 PMC4873725

[18] Boeije H Willis G. The cognitive interviewing reporting framework (CIRF). Methodology 2013; 9: 87–95.

[19] Murtagh FE Addington-Hall JM Higginson IJ. The value of cognitive interviewing techniques in palliative care research. Palliat Med 2007; 21: 87–93. 17344256 10.1177/0269216306075367

[20] Collins D. Cognitive interviewing practice. London: Sage, 2015.

[bibr21-02692163241248735] Tourangeau R. Cognitive sciences and survey methods. In: National Research Council; Division of Behavioral and Social Sciences and Education; Commission on Behavioral and Social Cognitive aspects of survey methodology: building a bridge between disciplines. Washington DC: National Academy Press, 1984, pp.73–101.

[bibr22-02692163241248735] Willis G Schechter S Whitaker K. A comparison of cognitive interviewing, expert review, and behavior coding: what do they tell us? Am Stat Assoc 1999.

[23] Ritchie J Lewis J McNaughton-Nicholls C , et al Qualitative research practice: A guide for social science students and researchers. London: Sage, 2014.

[24] Fraser L Gibson-Smith D Jarvis S , et al ‘Make every child count’ estimating current and future prevalence of children and young people with life-limiting conditions in the United Kingdom. Palliat Med 2021; 35(9): 1641–1651. 33323043 10.1177/0269216320975308 PMC8532217

[25] Hain R Devins M Hastings R , et al Paediatric palliative care: development and pilot study of a 'Directory' of life-limiting conditions. BMC Palliat Care 2013; 12: 43. 24330676 10.1186/1472-684X-12-43 PMC4029745

[26] Arbuckle R Abetz-Webb L. “Not just little adults”: qualitative methods to support the development of pediatric patient-reported outcomes. Patient 2013; 6: 143–159. 23912695 10.1007/s40271-013-0022-3

[27] Matza LS Swensen AR Flood EM , et al Assessment of health-related quality of life in children: a review of conceptual, methodological, and regulatory issues. Value Health 2004; 7: 79–92. 14720133 10.1111/j.1524-4733.2004.71273.x

[28] Borgers N De Leeuw ED Hox J. Children as respondents in survey research: cognitive development and response quality. Bull Methodol Sociol 2000; 66: 60–75.

[29] Halstead P Arbuckle R Marshall C , et al Development and content validity testing of patient-reported outcome items for children to self-assess symptoms of the common cold. Patient 2020; 13: 235–250. 31858430 10.1007/s40271-019-00404-8 PMC7075834

[30] Zaman B Vanden Abeele V De Grooff D. Measuring product liking in preschool children: an evaluation of the smileyometer and this or that methods. Int J Child Comput Interact 2013; 1: 61–70.

[bibr31-02692163241248735] Mellor D Moore KA. The use of likert scales with children. J Pediatr Psychol 2014; 39: 369–379. 24163438 10.1093/jpepsy/jst079

[32] Read J Fine K . Using survey methods for design and evaluation in child computer interaction. Workshop on Child Computer Interaction: Methodological Research at Interact 2005.

[33] Rebok G Riley A Forrest C , et al Elementary school-aged children’s reports of their health: a cognitive interviewing study. Qual Life Res 2001; 10: 59–70. 11508476 10.1023/a:1016693417166

[34] Reggio C Mowbray C Waldron MK , et al “It can be hard but it’s not bad”: three questions to solicit caregiver perceptions of benefits and burdens to participating in pediatric palliative care research. J Palliat Med 2021; 24: 1641–1649. 33902327 10.1089/jpm.2020.0618 PMC9022129

[35] Weaver MS Mooney-Doyle K Kelly KP , et al The benefits and burdens of pediatric palliative care and end-of-life research: a systematic review. J Palliat Med 2019; 22: 915–926. 30835596 10.1089/jpm.2018.0483 PMC6755658

[36] Koch KD Jones BL. Supporting parent caregivers of children with life-limiting illness. Children 2018; 5: 20180626. 10.3390/children5070085 PMC6069074 29949926

