# Supplementary material for: Cognitive testing of the Children’s Palliative Outcome Scale (C-POS) with children, young people and their parents/carers
Source: Palliat Med. 2024 May 6;38(6):644–59. doi: 10.1177/02692163241248735 (PMC11158001; doi:10.1177/02692163241248735)
Supplement: sj-docx-1-pmj-10.1177_02692163241248735 – Supplemental material for Cognitive testing of the Children’s Palliative Outcome Scale (C-POS) with children, young people and their parents/carers [file sj-docx-1-pmj-10.1177_02692163241248735.docx]

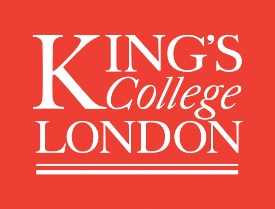
**Interview Guide for Cognitive Interview**

**Objective:** To explore the cognitive processes used by respondents when reading, interpreting and responding to items on the C-POS.

**Consent and demographics:**

- Hi my name is …. Thank you for agreeing to talk to me today.
- Have you had a chance to read the information sheet about the study?
- As the sheet says, we have developed a questionnaire asking about outcomes for parents/carers of children and young people with a serious illness. The aim of this interview is to see if the questions are understood in the way we intended, and whether the way we have presented the answers is clear. It doesn’t matter if they are not, because we have time to improve the questions. What you tell us will help us to improve the questionnaire.
- We are also keen to find out whether you think there are any important questions missing or any that aren’t relevant or useful. There are no right or wrong answers to any of the questions.
- We will be recording the interview and anything that you tell us is confidential.
- If you want me to stop the interview or stop recording at any time, then just let me know. It’s fine if you need to take a break or don’t want to continue.
- Go through assent and/or consent forms and sign. (Ask consenting person to complete and return either photo/scanned copy or post paper copy). **Please record conversation as separate audio-file.**
- Ask participant demographic questions (*share screen and fill out separate sheet*).

**Introduction to interview:**

- So, I’m going to show you some questions (either on the screen/on paper) and I would like you to read & answer them one at a time
- We will stop and talk about each question before moving onto the next.
- As you answer I’d like you to try to ‘think out loud’ as you read and answer the question.
- Is it ok if we practice with a question first? I’ll demonstrate and then you can have a go.
  - [Demonstrate dummy question.] *Either for:*
    - *Children:* ‘Can you tell me what you have in the room where you sleep?’
    - *Adults/Young people*: ‘How many windows do you have in your home?’
    - How about you try with the same question?
  - Remember to try to ‘think out loud’ as you read and answer the questions.
  - [Ask prompts as needed for dummy question.]
  - Thank them for responding and give feedback.
- During the interview I will also ask you some more specific things about each question.
- Apologies if the questions get repetitive
- In this study we are less interested in your answers to the questions, but how you arrive at the answers – what you think the question means, and the things you were thinking about when you chose your answer. There are no right or wrong answers.
- You can tell me any thoughts or views you might have about the questions as we go along.
- Ask permission to start recording

--------------------------------------------------- START RECORDING --------------------------------------------

General:

- What were you thinking about when you answered that question?
- (*If there was any hesitation, follow-up*) – You seemed to hesitate – can I ask what were you thinking about then?

Comprehension: **What does the respondent believe the question to be asking?**

- What does the question mean to you, in your own words?
- What does the word/term XXXXXX mean to you? (*Refer to corresponding sheet for flagged words, OR raise if certain words seem problematic*)
- How easy or difficult was it to understand this question?
- (*If problem*) How would you change this question?

Retrieval: **Could they recall the information required by the question? Was the time frame suitable?**

- How easy or difficult was it to remember your/your child’s experience when answering this question?
- Was it easy or difficult to think about [“*the* *past week” / “yesterday or today”*] when answering this question?
- Would there be a different time period that would be easier to understand?

Judgement: **Is the respondent able to make an evaluation based on the information recalled?**

- How did you arrive at your answer to that question?
- Was that easy or hard to arrive at your answer? Why do you say that?
- How did you choose your answer?
- How sure are you of the answer to this question?

***(NOTE****: Consider using easier version for 8-12yrs and 13-18yrs if they struggle with a 5-point response format.)*

Response: **Is the respondent able to map their internally generated answer to a response option?**

- How did you choose your answer to this question?
- Was it hard or easy to select an answer from the options given?
- Did all options make sense for this question?
- What do you think the difference is between (chosen option – e.g. “*sometimes*”) and (option next to it – e.g. “*often*”)?
- If there are different response formats given – ask which format they preferred and why? Which was easiest to use?

**Other:**

- Is there anything else you would like to say about this question? / The questionnaire as a whole?
- Did you find any of the questions upsetting? / embarrassing? / inappropriate?
- Are there any questions that you would leave out of this questionnaire?
- Are there any questions that you would add to this questionnaire?

---------------------------------------------- THANKS + STOP RECORDING -------------------------------------
